# Elevated APE1/Ref-1 Levels of Synovial Fluids in Patients with Rheumatoid Arthritis: Reflection of Disease Activity

**DOI:** 10.3390/jcm10225324

**Published:** 2021-11-16

**Authors:** In Seol Yoo, Yu-Ran Lee, Seong Wook Kang, Jinhyun Kim, Hee-Kyoung Joo, Su-Jin Yoo, Chan Keol Park, Ha-Reum Lee, Ji Ah Park, Byeong-Hwa Jeon

**Affiliations:** 1 Division of Rheumatology, Department of Internal Medicine, Chungnam National University Sejong Hospital, 20 Bodeum 7-ro, Sejong 30099, Korea; cptmiller@cnuh.co.kr (I.S.Y.); plutocys@cnuh.co.kr (C.K.P.); 2 Department of Internal Medicine, College of Medicine, Chungnam National University, 266 Munhwa-ro, Jung-gu, Daejeon 35015, Korea; kangsw@cnuh.co.kr (S.W.K.); jkim@cnuh.co.kr (J.K.); sujin428@cnuh.co.kr (S.-J.Y.); 3 Research Institute for Medical Sciences, College of Medicine, Chungnam National University, 266 Munhwa-ro, Jung-gu, Daejeon 35015, Korea; lyr0913@cnu.ac.kr (Y.-R.L.); hkjoo79@cnu.ac.kr (H.-K.J.); hareum_lee@daum.net (H.-R.L.); pjiah13@hanmail.net (J.A.P.); 4 Department of Physiology, College of Medicine, Chungnam National University, 266 Munhwa-ro, Jung-gu, Daejeon 35015, Korea; 5 Division of Rheumatology, Department of Internal Medicine, Chungnam National University Hospital, 282 Munhwa-ro, Jung-gu, Daejeon 35015, Korea

**Keywords:** apurinic/apyrimidinic endonuclease 1/redox factor-1, rheumatoid arthritis, DAS28, ELISA

## Abstract

There is growing evidence that apurinic/apyrimidinic endonuclease 1/redox factor-1 (APE1/Ref-1) regulates inflammatory responses. Rheumatoid arthritis (RA) is an autoimmune disease, which is characterized with synovitis and joint destruction. Therefore, this study was planned to investigate the relationship between APE1/Ref-1 and RA. Serum and synovial fluid (SF) were collected from 46 patients with RA, 45 patients with osteoarthritis (OA), and 30 healthy control (HC) patients. The concentration of APE1/Ref-1 in serum or SF was measured using the sandwich enzyme-linked immunosorbent assay (ELISA). The disease activity in RA patients was measured using the 28-joint disease activity score (DAS28). The serum APE1/Ref-1 levels in RA patients were significantly increased compared to HC and OA patients (0.44 ± 0.39 ng/mL for RA group vs. 0.19 ± 0.14 ng/mL for HC group, *p* < 0.05 and vs. 0.19 ± 0.11 ng/mL for OA group, *p* < 0.05). Likewise, the APE1/Ref-1 levels of SF in RA patients were also significantly increased compared to OA patients (0.68 ± 0.30 ng/mL for RA group vs. 0.31 ± 0.12 ng/mL for OA group, *p* < 0.001). The APE1/Ref-1 concentration in SF of RA patients was positively correlated with DAS28. Thus, APE1/Ref-1 may reflect the joint inflammation and be associated with disease activity in RA.

## 1. Introduction

Rheumatoid arthritis (RA) is an autoimmune disease characterized by synovitis, joint destruction, and other damage caused by systemic inflammation [1]. Although the pathogenesis of RA is complicated, chronic synovitis is the key feature of RA. The synovium of RA exhibits the following specific pathological features: (1) proliferation of synoviocytes; (2) infiltration of various immune cells; (3) neo-angiogenesis; (4) increased expression of adhesion modules on cell surfaces; and (5) expression of various cytokines and matrix-degrading enzymes [2]. The infiltrated immune cells within the synovium are mostly mononuclear cells such as T lymphocytes, B lymphocytes, plasma cells, macrophages, dendritic cells, and mast cells. Various cytokines are mainly secreted from these cells, which leads to the recruitment of inflammatory cells and joint destruction. Among these cytokines, tumor necrosis factor (TNF)-α and interleukin (IL)-1 are key proinflammatory cytokines that are mainly secreted by macrophages and induce the expression of IL-6, IL-8, IL-10, adhesion molecules such as vascular cell adhesion molecule-1 (VCAM-1), and matrix metalloproteinases (MMPs) [2]. These inflammatory mediators accelerate bone erosion, cartilage destruction, and systemic inflammatory responses (including anemia, fatigue, and cardiovascular disease) [1,2]. Inhibitors of these cytokines (especially TNF-α and IL-6 inhibitors) have already been implicated in the treatment of RA [3,4].

Apurinic/apyrimidinic endonuclease 1/redox factor-1 (APE1/Ref-1) is an enzymatic protein with redox activity and endonuclease activity. It can recognize the DNA damage and has repair activity. In addition, APE1/Ref-1 can modulate the redox status and various transcription factors (TFs) [5,6]. APE1/Ref-1 activates numerous TFs, such as c-Jun, p53, nuclear factor kappa B (NF-κB), activator protein-1 (AP-1), hypoxia-inducible factor 1α (HIF-1α), and paired box gene 8 (PAX8), which regulate diverse cellular processes involving cell growth, signaling, and inflammation [7,8,9,10,11]. APE1/Ref-1 has three types of subcellular localization (nuclear, cytoplasmic, and extracellular) and exhibits different roles accordingly. Generally, APE1/Ref-1 is located in the nucleus of most cells in basal conditions [12]. However, the localization of APE1/Ref-1 is dynamically regulated by oxidative stress and DNA damage, which leads to cytoplasmic translocation or extracellular secretion [13]. The key role of APE1/Ref-1 as a proinflammatory mediator has been emphasized in numerous studies. Nath et al. suggested that the extracellular APE1/Ref-1 increases the expression and secretion of IL-6 and activates NF-κB [14]. On the other hand, the anti-inflammatory actions of extracellular APE1/Ref-1 have been reported in recent studies. Extracellular APE1/Ref-1 downregulated the lipopolysaccharide (LPS)-induced proinflammatory cytokines and chemokines [15]. Moreover, APE1/Ref-1 suppressed the monocyte adhesion and VCAM-1, through a nitric oxide synthase (NOS)-dependent mechanism in endothelial cells [16]. Hall et al. found that the upregulation of APE1/Ref-1 promotes endothelial cell survival through TNF-α and hypoxia-induced NF-κB-independent/dependent signaling pathways [17].

As mentioned above, TNF-α, IL-1, IL-6, and VCAM-1 are key inflammatory mediators in the pathogenesis of RA and the target molecules of extracellular APE1/Ref-1 in inflammatory conditions. Therefore, extracellular APE1/Ref-1 might have crucial functions in the pathogenesis of RA. However, there have been no studies on the relationship between serum or synovial fluid (SF) APE1/Ref-1 and RA. The aim of this study was to demonstrate the usefulness of APE1/Ref-1 for the diagnosis of disease activity in RA by investigating the serum and SF level of APE1/Ref-1 in patients with RA and osteoarthritis (OA).

## 2. Materials and Methods

### 2.1. Patients

Forty-six RA patients, forty-four OA patients, and thirty healthy control (HC) patients were enrolled. The health control samples were obtained during the routine health checkups of healthy adults without underlying diseases. RA patients fulfilled the 1987 American College of Rheumatology (ACR) classification criteria or 2010 ACR/European League Against Rheumatism (EULAR) classification criteria [18,19]. The data collected were as follows: age, gender, body mass index (BMI), patient global assessment (PtGA), tender joint count (TJC), swollen joint count (SJC), C-reactive protein (CRP), erythrocyte sedimentation rate (ESR), anticyclic citrullinated peptide antibody (ACPA), rheumatoid factor (RF), and current medication. The disease activity in RA patients was assessed using the 28-joint disease activity score (DAS 28), using PtGA, TJC, SJC, ESR, and CRP (DAS28-ESR/DAS28-CRP) [20]. OA patients fulfilled the ACR criteria for knee OA [21]. OA patients were excluded from the study if they had received intra-articular injections in the joint within 3 months or systemic glucocorticoid therapy. This study protocol was reviewed and approved by the Institutional Review Board of Chungnam National University Hospital (2014-08-043) and performed according to the Declaration of Helsinki. All subjects provided their informed written consent before participation.

### 2.2. Sample Preparation

Blood samples were drawn into EDTA-treated vacuum tubes (BD, Franklin Lakes, NJ, USA) during treatment procedures. Serum was separated by centrifugation at 2000× *g* for 10 min. SF was collected in the arthrocentesis procedure from all patients with OA and RA. To remove cells and debris in the SF, the supernatant was collected by centrifugation at 2000× *g* for 15 min. The viscosity of SF was similar in OA and RA patient groups. Serum and SF were used undiluted for analysis and were stored as 0.5 mL aliquots at −80 °C until analysis.

We obtained 30 serum samples and 30 SF samples (16 paired samples from the same patients) from 45 OA patients, and 29 serum samples and 31 SF samples (13 paired samples from the same patients) from 46 RA patients. SF samples from patients with RA and OA were collected during diagnostic and therapeutic arthrocenteses (for example, intra-articular glucocorticoid or hyaluronic acid injection) of a knee joint. Control serum samples were randomly selected from 30 healthy subjects.

### 2.3. Quantification of APE1/Ref-1 in Serum and Synovial Fluids

The concentration of APE1/Ref-1 in each serum and SF sample was analyzed quantitatively using a sandwich enzyme-linked immunosorbent assay (ELISA) according to the manufacturer’s instructions (Mediredox, Daejeon, Korea). The serum, SF sample, and standard were each added to a coated 96-well microplate with the capture antibody, and diluent was added to the blank. The absorbance was measured at 450 nm with an automatic microplate reader (Glomax, Promega, Madison, WI, USA). Each sample was measured in duplicate and mean values were determined. A standard curve was established using recombinant human APE1/Ref-1 protein (rhAPE1/Ref-1, MediRedox, Daejeon, Korea). The rhAPE1/Ref-1 protein was serially diluted (2-fold) in diluent and used at 0.078–5 ng/mL. The minimum detection range of human APE1/Ref-1 ELISA kit was 0.078 ng/mL.

### 2.4. Statistical Analysis

The sample size was determined by a preliminary pilot study and recommendation of statistical experts in clinical research. A paired or unpaired *t*-test or analysis of variance (ANOVA) followed by Bonferroni’s multiple comparison test was used to compare the level of APE1/Ref-1 between groups. The correlation of clinical characteristics and APE1/Ref-1 levels were analyzed by Pearson’s correlation analysis. The disease activity of RA patients was subdivided and analyzed according to the following criteria: low (DAS28 ≤ 3.2), moderate (3.2 < DAS28 ≤ 5.1), and high (DAS28 > 5.1) [20]. To achieve remission, the RA patients had to have a DAS28 < 2.6 [20]. The level selected for statistical significance was *p* < 0.05. Receiver operating characteristic (ROC) curve analysis was used to calculate the sensitivity and specificity of APE1/Ref-1 when detecting OA and RA. The data were analyzed with SPSS (IBM SPSS Statistics) version 20.0 for Windows (SPSS Inc., Chicago, IL, USA).

## 3. Results

### 3.1. Patient Characteristics

Among 91 patients, 46 were RA patients. HC subjects who had not been diagnosed with any arthritis were used as controls for patients. As the HC group was randomly selected in a biobank, we could not obtain information on BMI, ESR, and CRP. There were no statistical differences in demographic characteristics (such as age or gender) between the RA patients and OA patients. In addition, BMI did not differ between the OA and RA groups. Twelve patients were first diagnosed with RA at the time of the study. Nine patients achieved remission (DAS28 < 2.6). The clinical characteristics of the patients are presented in Table 1.

### 3.2. Elevated Concentration of APE1/Ref-1 in RA Patients

**Figure 1:**
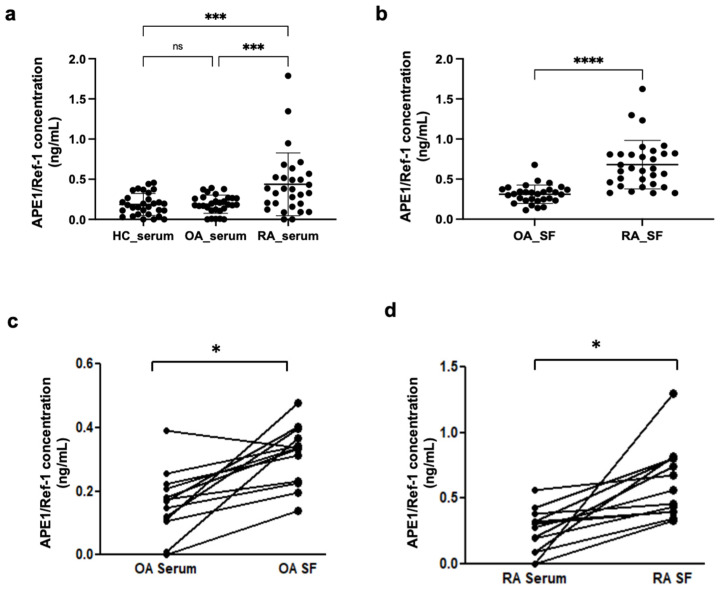
The concentration of apurinic/apyrimidinic endonuclease 1/redox factor-1 (APE1/Ref-1) was increased in synovial fluid (SF) of rheumatoid arthritis (RA) patients. (**a**) The serum levels of APE1/Ref-1 in patients with RA (*n* = 29) were significantly elevated compared to those of the healthy control (HC) (*n* = 30) and osteoarthritis (OA) patient groups (*n* = 30). (**b**) The RA patient group (*n* = 31) had a significantly higher APE1/Ref-1 concentration in their SF than that of the OA patient group (*n* = 30). Dot plots represent the mean; bars represent the SD. *** *p* < 0.001, **** *p* < 0.0001 vs. RA group based on one-way ANOVA analysis followed by Bonferroni’s multiple comparison test and unpaired *t*-test. (**c**,**d**) Significant correlations were found between serum and SF APE1/Ref-1 levels in paired OA samples (*n* = 16) (**c**) and RA samples (*n* = 13). (**d**) Statistical significance was determined by paired *t*-test. * *p* < 0.05 vs. OA_serum or RA_serum.

First, we compared the serum and SF concentrations of APE1/Ref-1 in OA and RA groups. There was no statistical difference of serum APE1/Ref-1 concentration between the HC and OA patient groups (0.19 ± 0.14 ng/mL for HC vs. 0.19 ± 0.11 ng/mL for OA) (Figure 1a). The serum concentrations of APE1/Ref-1 in the RA patient group were significantly increased compared to those of the HC group and OA patient group (0.44 ± 0.39 ng/mL for RA group vs. 0.19 ± 0.14 ng/mL for HC, *p* < 0.001 and 0.19 ± 0.11 ng/mL for OA group, *p* < 0.001) (Figure 1a). Furthermore, the RA patient group had a significantly higher APE1/Ref-1 level in their SF than that of the OA patient group (0.68 ± 0.31 ng/mL for RA group vs. 0.31 ± 0.12 ng/mL for OA group, *p* < 0.001) (Figure 1b). Additionally, paired samples of serum and SF from patients with OA and RA were examined regarding the relationship between systemic and target tissue levels of APE1/Ref-1. Paired SF and serum samples from 13 patients with RA and 16 with OA were collected during diagnostic and therapeutic arthrocenteses of knee joints. Significant correlations were found between SF and serum APE1/Ref-1 levels in paired OA samples (r = 0.626; *p* = 0.002) (Figure 1c) and RA samples (r = 0.569; *p* = 0.0018) (Figure 1d).

### 3.3. The Receiver Operating Characteristic (ROC) Curve of APE1/Ref-1 Levels in RA Patients

**Figure 2:**
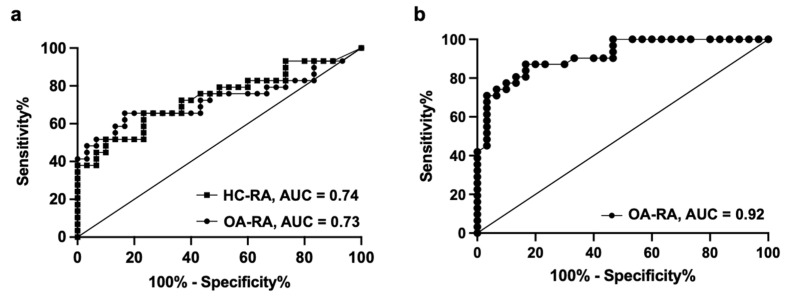
The receiver operating characteristic (ROC) curve of apurinic/apyrimidinic endonuclease 1/redox factor-1 (APE1/Ref-1) levels in serum and synovial fluid (SF) of rheumatoid arthritis (RA) patients. (**a**) The ROC curve from serum samples of patients with osteoarthritis (OA) and RA resulted in an area under the curve (AUC) of 0.73, and that of healthy control (HC) patients and RA patients resulted in an AUC of 0.74. (**b**) The ROC curve from SF samples of patients with OA and RA resulted in an AUC of 0.92.

The ROC curve from the serum samples of patients with OA and RA resulted in an area under the curve (AUC) of 0.73 (95% confidence interval, 0.591 to 0.864). On this basis, the optimal combination of sensitivity and specificity was determined to be a sensitivity of 65% and a specificity of 67%, using a cutoff value of 0.24 ng/mL (serum) (Figure 2a). Additionally, we analyzed the ROC curve from serum samples of HC patients and patients with RA, which resulted in an area under the curve (AUC) of 0.74 (95% confidence interval, 0.608 to 0.867). On this basis, the optimal combination of sensitivity and specificity was determined to be a sensitivity of 66% and a specificity of 70%, using a cutoff value of 0.232 ng/mL with HC patients (Figure 2a). The ROC curve from SF samples of patients with OA and RA resulted in an AUC of 0.92 (95% confidence interval, 0.847 to 0.983) (Figure 2b). The optimal combination of sensitivity and specificity was determined to be a sensitivity of 81% and a specificity of 83%, using a cutoff value of 0.40 ng/mL (SF).

### 3.4. Correlation between Synovial APE1/Ref-1 and RA Disease Activity

**Figure 3:**
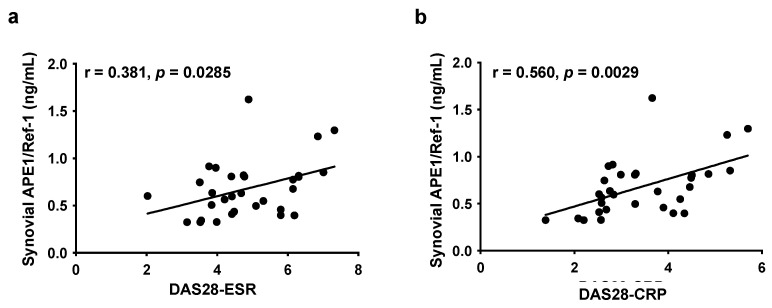
Correlation between synovial APE1/Ref-1 levels and rheumatoid arthritis (RA) disease activity; (**a**,**b**) 28-joint disease activity score (DAS 28), using ESR and CRP (DAS28-ESR/DAS28-CRP). Statistical significance was determined by Pearson’s correlation analysis.

To address whether synovial APE1/Ref-1 levels correlate with RA disease activity, we performed Pearson’s correlation analysis with several clinical parameters (DAS28-ESR or DAS28-CRP). DAS28-ESR showed a weak positive correlation with SF concentration of APE1/Ref-1 (r = 0.381, *p* < 0.05) (Figure 3a). However, DAS28-CRP was significantly correlated with the SF concentration of APE1/Ref-1 (r = 0.560, *p* < 0.01) (Figure 3b).

**Figure 4:**
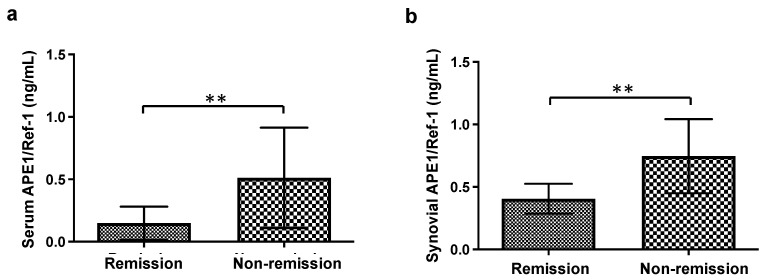
The comparison of APE1/Ref-1 levels between the remission group (DAS 28 < 2.6) and the nonremission group (DAS 28 > 2.6). (**a**) The serum APE1/Ref-1 concentration of the remission group (*n* = 6) and the nonremission group (*n* = 23). (**b**) The synovial APE1/Ref-1 concentration of the remission group (*n* = 6) and the nonremission group (*n* = 25). Columns represent the mean; bars represent the SD. ** *p* < 0.01, vs. remission according to an unpaired *t*-test.

The remission criteria define the absence of disease activity. DAS28 = 2.6 is used as a cutoff value that distinguishes remission and nonremission [20]. Thus, we evaluated the APE1/Ref-1 levels of SF in the remission group (DAS 28 < 2.6) and the nonremission group (DAS28 > 2.6). As shown in Figure 4, the serum and synovial concentrations of APE1/Ref-1 in the remission group (DAS 28 < 2.6) were significantly lower than that in the nonremission group (DAS28 > 2.6) (serum APE1/Ref-1: 0.15 ± 0.13 ng/mL for the remission group vs. 0.51 ± 0.40 ng/mL for the nonremission group, *p* < 0.01; synovial APE1/Ref-1: 0.41 ± 0.12 ng/mL for the remission group vs. 0.75 ± 0.30 ng/mL for the nonremission group, *p* < 0.01). In these regards, the serum or SF concentrations of APE1/Ref-1 in the RA patient group was more closely related to parameters of disease activity.

**Figure 5:**
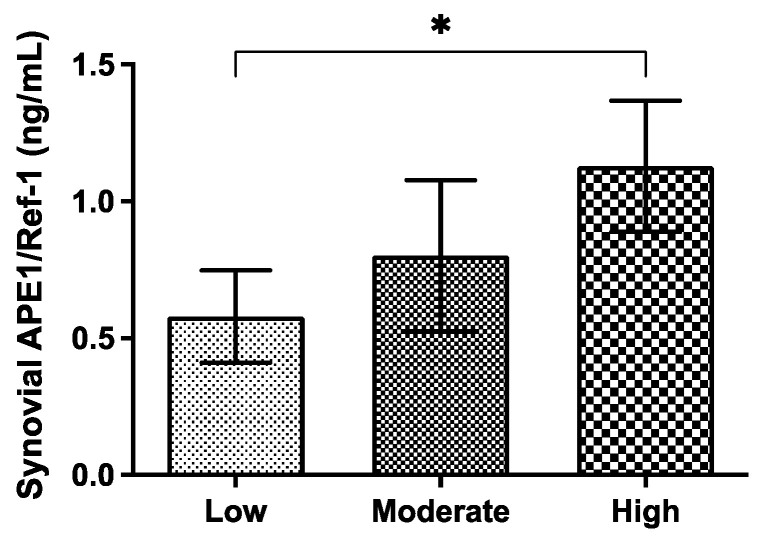
Synovial APE1/Ref-1 levels according to disease activity categories. The synovial APE1/Ref-1 concentrations were associated with disease activity categories. Columns represent the mean; bars represent the SD. * *p* < 0.05 vs. low based on one-way ANOVA analysis followed by Bonferroni’s multiple comparison test.

Additionally, the disease activity of RA patients was analyzed with the following categories: low (DAS28-CRP ≤ 3.2), moderate (3.2 < DAS28-CRP ≤ 5.1), and high (DAS28-CRP > 5.1) [20]. There was no significant correlation between the serum concentration of APE1/Ref-1 and disease activity categories. On the other hand, the concentration of APE1/Ref-1 in SF was more significantly increased in the high disease activity group than in the low disease activity group (Figure 5).

## 4. Discussion

This is the first study to investigate the change of APE1/Ref-1 levels in RA. In this study, we demonstrated that serum and synovial APE1/Ref-1 levels are higher in patients with RA, and that they are correlated with disease activity. Synovial APE1/Ref-1 had a high diagnostic accuracy for differentiation of RA from OA (AUC = 0.92, sensitivity—81%, specificity—83%, cutoff of 0.40 ng/mL).

APE1/Ref-1 is 37 kDa with a multifunctional protein and is mainly located in cell nuclei [22]. In response to various factors (for example, reactive oxygen species/reactive nitrogen species (ROS/RNS), and hypoxia), APE1/Ref-1 is involved in DNA base excision repair to maintain genomic integrity and acts as a reducing coactivator of many TFs in controlling different cellular processes, such as apoptosis, and is involved in survival pathways [5,23]. Generally, APE1/Ref-1 is localized in nuclei, but its localization is regulated in response to stress. This results in cytoplasmic/mitochondrial translocation or extracellular release [12,13]. APE1/Ref-1 exhibits diverse biological functions according to its subcellular localization. Notably, extracellular APE1/Ref-1 plays a key role in the regulation of inflammation. Park et al. reported that treatment with recombinant human APE1/Ref-1 protein inhibited TNF-α-induced VCAM-1 expression in human umbilical vein endothelial cells [24]. Joo et al. also demonstrated that the expression of TNF-α-induced VCAM-1 in endothelial cells and LPS-induced cyclooxygenase-2 (COX-2) in Raw264.7 cells was inhibited by extracellular APE1/Ref-1 and that this inhibitory effect was neutralized with an anti-APE1/Ref-1 antibody [15].

Furthermore, APE1/Ref-1 regulated the high-mobility group box 1 (HMGB1)-mediated inflammatory response [25]. HMGB1 is released by necrotic cells, monocytes, and macrophages after stimulation with endotoxin, TNF-α, and IL-1. Extracellular HMGB1 functions as a proinflammatory cytokine in a Toll-like receptor- (TLR) and receptor for advanced glycation end-products (RAGE)-dependent manner in severe sepsis and autoimmune diseases. The cytoplasmic APE1/Ref-1 attenuated the upregulation of ROS generation, cytokine secretion, and COX-2 expression by monocytes and macrophage-like THP-1 cell lines. HMGB1 facilitates the release of proinflammatory cytokines such as TNF-α, IL-1α, IL-1β, and IL-6 from monocytes. However, APE1/Ref-1 completely downregulated these cytokines through nicotinamide adenine dinucleotide phosphate (NADPH) oxidase activation and ROS generation in THP-1 cells [25]. In addition, the phosphorylation of mitogen-activated protein kinase (MAPK) facilitates the production of TNF-α and IL-6 in monocytes. The HMGB1-induced activation of p38 MAPK and c-Jun N-terminal kinase (JNK) was strongly downregulated by APE1/Ref-1. In addition, macrophages secrete HMGB1 via the TLR ligand, which acts as a proinflammatory cytokine. APE1/Ref-1 inhibited TLR/LPS-mediated extracellular release of HMGB1 in THP-1 cells [25]. Notably, HMGB1 has been associated with the pathogenesis of RA [26,27,28]. Taniguchi et al. confirmed that the synovial concentrations of HMGB1 increased in the patients with RA and HMGB1 was abundantly expressed in synovial tissue [27]. In addition, the serum concentrations of HMGB1 were elevated in patients with RA and correlated with DAS28 [28].

In addition to our study, there is a study on the role of APE1 in autoimmune diseases. Lee et al. reported that epidermal APE1/Ref-1 expression is significantly higher in psoriatic lesions than in a normal epidermis. They found that TLR2-mediated inflammatory mediators, including TNF-α, CXCL8, and LL-37, were overexpressed at the psoriatic lesions. APE1 silencing downregulated TNF-α, CXCL8, and LL-37 in HaCaT cells and human primary keratinocytes [29]. Thus, APE1/Ref-1 acts as a key regulator of epidermal hyperplasia and inflammation in psoriasis.

In numerous studies, APE1/Ref-1 has been demonstrated as a serological biomarker for cardiovascular disease and cancer [30,31,32,33]. The diagnosis of RA is quite difficult because there are no definite biomarkers. RF is detected in the majority of patients with RA, and included in the 1987 ACR classification criteria and ACR/EULAR classification criteria [18,19]. However, RF is also detected nonspecifically in various inflammatory conditions (e.g., Sjögren syndrome, sarcoidosis, hepatitis B and C infection, and tuberculosis) [34]. The false-positive rate of RF ranged from 1% to 5% in the general population [35]. ACPA is more specific than rheumatoid factors and is very useful in the early diagnosis and prognosis prediction of RA. However, a combination of RF and ACPA can only be detected in up to 80% of patients with RA [36]. To date, there are no definite diagnostic criteria or biomarkers for RA diagnosis.

Since Park et al. confirmed the extracellular APE1/Ref-1 in the plasma of endotoxemic rats, numerous studies have reported the value of extracellular APE1/Ref-1 as a serologic marker in several diseases [13,37]. APE1/Ref-1 can be secreted by two different pathways. In response to various inflammatory and exogenous stimuli (e.g., trichostatin A, LPS, testosterone, and coxsackievirus B3), APE1/Ref-1 is actively secreted from monocytes or macrophages and endothelial cells via ATP-binding cassette transporter 1 (ABCA1) or vesicle-mediated pathways [38]. It is not mediated by the endoplasmic reticulum (ER)-to-Golgi complex pathway. Lee et al. reported that brefeldin A (inhibitor of the ER-to-Golgi complex pathway) did not modulate the secretion of APE1/Ref-1. In necrotic or apoptotic cells, APE1/Ref-1 is passively released from the cytoplasm or nucleus [38]. Thus, there is growing evidence that extracellular APE1/Ref-1 is a useful serologic marker for inflammation or cell death.

In the present study, we demonstrated that RA patients have higher concentrations of APE1/Ref-1 in their sera, compared with HC or OA patients. Blood APE1/Ref-1 has been suggested as a biomarker of systemic inflammation in preclinical animal experiments [13]. In this study, there was no difference in serum APE1/Ref-1 concentrations between healthy normal subjects and OA patients. In osteoarthritis, CRP or ESR was within the normal range, and these results suggest that systemic inflammation may not exist in patients with OA. Although SF in OA was also used as a control group for SF in RA in this study, it is clear that inflammation (e.g., synovitis, infiltration of mononuclear cells, overexpression of inflammatory mediators) is present in OA joints. Nevertheless, the high concentrations of synovial APE1/Ref-1 in RA suggest that there is more severe synovial inflammation in RA joints than in OA joints. These results also demonstrate that APE1/Ref-1 reflects the degree of inflammation of the target organ. Furthermore, the disease activity parameters of RA (especially DAS28-CRP) were positively correlated with the SF concentration of APE1/Ref-1. DAS28 is the most commonly used disease activity assessment tool in RA [20]. The ACR and EULAR officially recommended the use of DAS28 in clinical trials and daily clinical practice [20]. Although recent studies reported high levels of concordance between DAS28-CRP and DAS28-ESR, discrepancies in classification into high and moderate disease activity have been reported. Notably, ESR can be influenced by numerous factors, such as age, gender, anemia, plasma viscosity, and red blood cell (RBC) dysmorphism. In this study, the synovial concentration of APE1/Ref-1 in the high disease activity group (DAS28 > 5.1) was significantly higher than that in the low disease activity group (2.6 < DAS28 < 3.1). Thus, our studies suggest that APE1/Ref-1 might reflect the degree of disease activity in the joints of RA patients.

This study has some limitations. This work involved only a small sample of RA patients. Nonetheless, we confirmed that the sample size had sufficient statistical power. The risk and severity of RA are strongly associated with shared epitope-coding human leukocyte antigen (HLA)-DRB1 alleles. The shared epitope increases the likelihood of an earlier disease onset, developing more severe bone erosions, and ACPA positivity. Moreover, it was reported that the DRB1*04 group showed higher ACPA titers than the DRB1*01 group in RA patients [39]. However, we could not analysis the HLA-DR subtyping.

Moreover, we collected both serum and SF samples from each group with paired patients at the same time. This is the strength of our study. A limitation of our study is that there is in no normal control group of SF, as collecting SF from healthy adults presents ethical challenges. It was possible to obtain the blood of a healthy adult, but not the SF of a normal person. Therefore, SF in patient with OA, a noninflammatory disease, was used as a control for rheumatoid arthritis. There is also the issue that, although we carefully assessed relevant medical records, we could not control other possible factors that could affect APE1/Ref-1 concentrations.

## 5. Conclusions

Taken together, the serum and synovial concentrations of APE1/Ref-1 in RA patients were higher than in OA patients. Moreover, the synovial APE1/Ref-1 levels in RA patients are well correlated with their disease activity (i.e., the DAS28-ESR/CRP). Therefore, APE1/Ref-1 can be a useful marker of synovial inflammation and, thereby, disease activity in RA or other synovial inflammatory diseases. If the correlation between APE1/Ref-1 and ACPA titer and shared epitope is investigated in the future, it will be a very useful biomarker for RA diagnosis and disease activity measurement.

## Figures and Tables

**Table 1:** General characteristics of healthy controls and patients with osteoarthritis or rheumatoid arthritis.

	HC (*n* = 30)	OA (*n* = 45)	RA (*n* = 46)
Age	53.9 ± 13.89	66.7 ± 10.8 **	64.6 ± 10.7 **
Sex (M:F)	1:1.5	1:3	1:2.5
BMI (kg/m^2^)	NA	24.9 ± 3.3	24.4 ± 3.6
ESR (mm/h)	NA	12.6 ± 7.5	61.4 ± 33.5 ^##^
CRP (mg/dL)	NA	0.2 ± 0.1	2.2 ± 2.0 **
Rheumatoid factor (+)	NA	0 (0%)	43 (93.5%)
Anti-cyclic citrullinated peptide (+)	NA	0 (0%)	45 (97.8%)
DAS28-ESR	NA	NA	4.6 ± 1.4
DAS28-CRP	NA	NA	3.2 ± 1.3

Data expressed as mean ± SD. Statistical significance was determined by *t*-test. ** *p* < 0.01, vs. HC, ^##^ *p* < 0.01, vs. OA. BMI: body mass index; CRP: C-reactive protein; DAS28: 28-joint disease activity score; ESR: erythrocyte sedimentation rate; HC: healthy control; NA: nonavailable; OA: osteoarthritis; RA: rheumatoid arthritis.
